# Allosteric induction of the CD4-bound conformation of HIV-1 Gp120

**DOI:** 10.1186/1742-4690-10-147

**Published:** 2013-12-05

**Authors:** Anna Roitburd-Berman, Gal Dela, Gilad Kaplan, George K Lewis, Jonathan M Gershoni

**Affiliations:** 1 Department of Cell Research and Immunology, George S. Wise Faculty of Life Sciences, Tel Aviv University, Tel Aviv, Israel; 2 The Institute of Human Virology, University of Maryland School of Medicine, Baltimore, MA USA; 3 Present address: Bio-Technology General Ltd., Kiryat Malachi, Israel

**Keywords:** HIV-1, Vaccine, gp120, CD4i, Phage display, Peptide

## Abstract

**Background:**

HIV-1 infection of target cells is mediated *via* the binding of the viral envelope protein, gp120, to the cell surface receptor CD4. This interaction leads to conformational rearrangements in gp120 forming or revealing CD4 induced (CD4i) epitopes which are critical for the subsequent recognition of the co-receptor required for viral entry. The CD4-bound state of gp120 has been considered a potential immunogen for HIV-1 vaccine development. Here we report on an alternative means to induce gp120 into the CD4i conformation.

**Results:**

Combinatorial phage display peptide libraries were screened against HIV-1 gp120 and short (14aa) peptides were selected that bind the viral envelope and allosterically induce the CD4i conformation. The lead peptide was subsequently systematically optimized for higher affinity as well as more efficient inductive activity. The peptide:gp120 complex was scrutinized with a panel of neutralizing anti-gp120 monoclonal antibodies and CD4 itself, illustrating that peptide binding does not interfere with or obscure the CD4 binding site.

**Conclusions:**

Two surfaces of gp120 are considered targets for the development of cross neutralizing antibodies against HIV-1; the CD4 binding site and CD4i epitopes. By implementing novel peptides that allosterically induce the CD4i epitopes we have generated a viral envelope that presents both of these surfaces simultaneously.

## Background

Infection of CD4 positive cells by HIV-1 is realized *via* a series of protein:protein interactions that escort the virus through specific “checkpoints” which include sequential recognition events of two cellular receptors with the viral envelope (trimeric gp120 + gp41). The binding of HIV-1 gp120 to cellular CD4 is the first of these critical steps [1,2], triggering conformational rearrangements in both proteins, forming and revealing CD4 induced (CD4i) epitopes [3-7]. These neo-epitopes have been demonstrated by the isolation of discriminating monoclonal antibodies (mAbs) that show a distinct preference [8-13] or an absolute stringent requirement [14-17] for the gp120:CD4 complex as compared to binding of either CD4 or gp120 alone. Subsequent binding of a second receptor, the chemokine receptors CCR5 or CXCR4 [18-20], becomes possible as a result of the stabilization and exposure of a specific CD4i epitope comprised of 4 anti-parallel beta strands of the gp120 outer domain referred to as the “bridging sheet” [9]. Following gp120:CCR5 interaction, further conformational rearrangements ensue leading to the assembly of the 6 helix bundle in gp41, juxtaposing the viral membrane to that of the cell facilitating their fusion [7,21,22]. As a result, the viral core enters the cellular cytoplasm and proceeds to infect the target cell.

Obviously, the two critical binding surfaces of gp120 are “strapped” – restricted in their ability to undergo substantial genetic variation [9,23]. These surfaces are compelled to conserve structural complementarity to their corresponding cellular receptors, CD4 and CCR5/CXCR4 respectively, so to ensure efficient binding. Consequently, the virus has evolved various strategies to reduce the accessibility of these functional, conserved surfaces in order to evade immune surveillance [24]. Nonetheless, mAbs that target the CD4 binding site (CD4bs) and CD4i epitopes are generated and not surprisingly, constitute hallmark components of broadly cross neutralizing (BCN) serum of those HIV-1 infected individuals that are able to keep the virus in check (e.g. natural viral suppressors) [25-31].

Hence, as the efforts to develop an effective prophylactic vaccine against HIV involve numerous strategies [32-34], one aspect of vaccine design becomes the attempt to focus the B-cell response towards the conserved CD4bs and CD4i epitopes. Native gp120 and trimeric envelope have evolved to suppress the immunogenicity of these sites. So the challenge is to create preferred more effective presentations of the viral envelope that better accentuate those conserved surfaces HIV-1 would otherwise conceal. One approach for this has been the idea of using the gp120:CD4 complex as a vaccine [14,35], thereby stabilizing the CD4-bound conformation of gp120 thus constitutively presenting its CD4i epitopes, although at the expense of occluding the CD4bs. Indeed, stabilization of the gp120:CD4 complexes either through chemical cross linking or by molecular genetic construction of gp120 linked directly to CD4 to create full length single chain (FLSC) gp120:CD4 has proven useful [35,36]. DeVico et al. demonstrated that SHIV-challenged rhesus macaques first immunized with cross-linked or FLSC gp120:CD4 complexes elicited high titers of CD4i Abs which correlated with lower blood and tissue-viremia, indicating that persistent presentation of CD4i epitopes in a vaccine could be beneficial [37].

Here we describe unique peptide modulators of gp120 that specifically interact with the viral envelope, elicit the CD4i epitopes recognized by defining antibodies but do so allosterically, i.e., without binding or obstructing the CD4bs. The peptide modulators bind monomeric as well as trimeric gp120 and lock the envelope in the preferred CD4-bound conformation while retaining a fully accessible CD4bs.

## Results

### Isolation of a novel gp120-binding peptide

**Figure 1:**
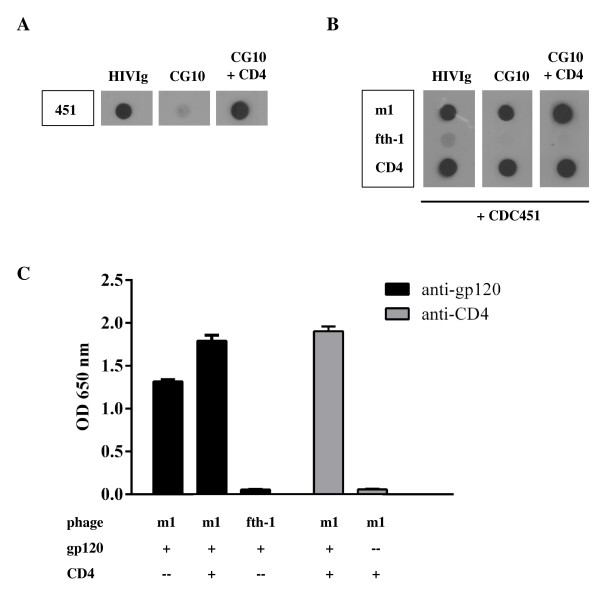
**The m1-phage binds gp120 and locks it into a CD4i conformation without interfering with CD4 binding. A.** Dot blot illustrating the stringent dependence of mAb CG10 on CD4. CDC451 gp120 was spotted onto nitrocellulose and detected with HIVIg, mAb CG10 or mAb CG10 in the presence of CD4. CG10 is strictly dependent on the presence of CD4 for gp120 binding. **B.** Equal amounts of phages displaying the m1-peptide, phages with no insert (fth-1) or CD4 were spotted to nitrocellulose filters. Filters were incubated with gp120 CDC451 or in blocking solution without gp120 (no signals developed in the absence of gp120, not shown) and bound gp120 was detected using HIVIg or the CD4i CG10 mAb. **C.** Equal amounts of phages displaying the m1-peptide or phages with no insert (fth-1) were captured on ELISA wells using an anti-M13 mAb. Wells were incubated with pre-formed gp120 CDC451:CD4 complex (stoichiometric molar ratio of 1:1), gp120 or CD4 alone, as indicated. Bound gp120 was detected using a biotinylated anti-gp120 9G3 mAb; bound CD4 was detected by a biotinylated anti-CD4 mAb CG9. The experiment was carried out in triplicate.

The HIV envelope undergoes conformational rearrangements upon association with CD4. These conformational changes can be monitored by the acquisition of binding of CD4i mAbs that are specific for the CD4-complexed gp120. CD4i mAbs can be divided into two categories; relaxed mAbs that bind gp120 albeit with a preference for the gp120:CD4 complex, as is the case for mAb 17b [9,11]; and stringent CD4i mAbs (e.g., CG10, 19e and N12-i15 [14-17,25,38-40]) that have an absolute strict requirement for bound CD4 before gp120 can be recognized. The objective of this study was to select a peptide that binds to the HIV-1 envelope and in doing so induces the *stringent* CD4i conformation, i.e., the ability to bind CG10 in the absence of CD4. For this, a random phage display peptide library (complexity = 5 × 10^9^) was screened using monomeric T-cell lab-adapted HIV-1 CDC451 gp120 as bait. Multiple rounds of biopanning of the phage display peptide library led to the isolation of a phage, designated m1 (amino acid sequence displayed: *C-DRRDLPQWAKRE-C*), which not only bound to gp120 but also enabled the binding of the stringent CD4i mAb CG10 [14,16,17,38,39] (Figure 1A and B).

A simple explanation of these results could be that m1-phage acts as a CD4-mimetic; binding gp120 at the CD4bs, inducing the corresponding conformational rearrangement. In order to test this hypothesis, the m1-peptide displaying phages were captured with an anti-M13 mAb and reacted with gp120 compared to gp120 mixed equi-molarly with soluble CD4 to generate a gp120:CD4 complex. Surprisingly, as shown in Figure 1C, CD4 did not compete for m1-phage binding to gp120. Quite the contrary, CD4 seemed to enhance the binding of m1-peptide for g120 while no affinity for CD4 alone could be detected (see Figure 1C). The fact that CD4 and m1-phage bound simultaneously to gp120 indicates that the two molecules have different and distinct epitopes on the HIV envelope.

The m1-peptide is thus a unique conformation-modulator of gp120 as it not only binds gp120 and drives the exposure of the CD4i epitopes but does so allosterically, i.e., without binding and occluding the binding site for CD4, as will be further substantiated below. Such a peptide might be valuable in the design of a gp120-based immunogen since it allows simultaneous exposure of the CD4i epitopes along with a functional CD4bs, which are both perceived to be targets of BCN antibodies [9,23,26,29-31,37,40-44].

### Optimization of the m1-peptide

**Figure 2:**
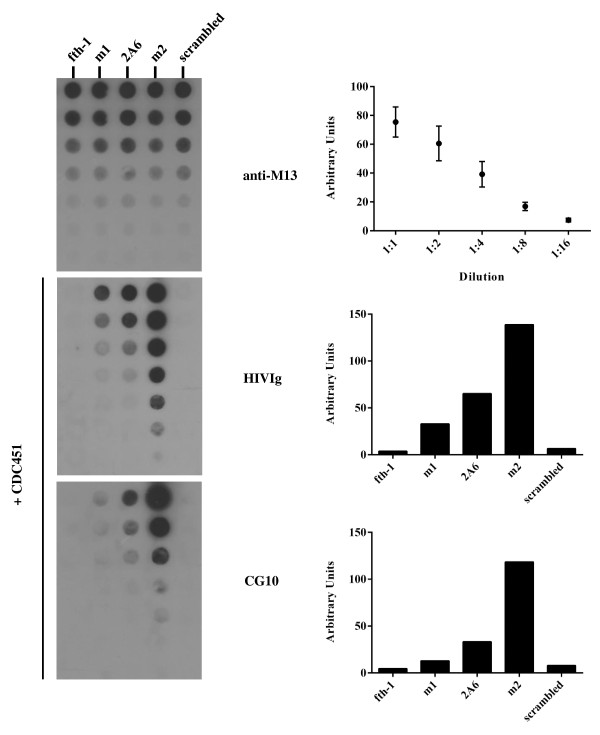
**Semi-quantitative dot blot analyses of m2-phage binding to gp120.** Equal amounts of 5 different phages were applied to nitrocellulose filters (two fold serial dilutions) and reacted with rabbit anti-M13 polyclonal sera, or gp120 CDC451 followed by HIVIg or CG10 mAb as indicated. As is illustrated in the top filter, the amount of phages at each dilution for each of the five different phages is similar and the ECL signal drops as the phages are diluted (see densitometric quantification in the histogram on the right and Methods). The binding of gp120 and detection with CG10 is enhanced for phage 2A6 (obtained in standard biopanning) and markedly improved for m2-phage (obtained in stringent biopanning) compared to phage m1, as is detected in the filters and their subsequent densitometric scans (histograms on the right) which were performed for both HIVIg and CG10 at dilution 1:4. Peptide sequences: m1 – *C-DRRDLPQWAKRE-C*; 2A6 – *C-DRRDLPQWAIRE-C*; m2- *C-DRRDLPDWAIRA-C*; scrambled – *C-DLWRIRADRAPD-C*; fth-1 – no insert.

In light of the potential importance of the m1-phage as an envelope conformation-modulator, efforts were made to optimize and isolate an improved, high affinity version of this peptide. For this we constructed a custom tailored, second generation library using biased random mutagenesis [45]. This method was developed to better exploit the size and complexity of random peptide phage display libraries. In principle, a lead peptide serves as a template for potential modification at every amino acid position which can assume any of the other 19 residues as compared to the “native” template (see Methods). Hence, using 3.3% contamination of the “other” phosphoramidites at each position, a library was produced containing 4 × 10^8^ m1-mutants which on average varied by 3–4 amino acids from the original m1-peptide. Screening of this library against gp120 CDC451, while applying standard or stringent biopanning conditions (see Methods), led to the isolation of gp120-specific peptides as are listed in Table 1. These peptides differed in their binding strength to gp120 CDC451 and in their ability to induce the CD4i conformation in gp120, as demonstrated in Figure 2, which shows the transition from the original m1-phage to a high affinity envelope binder, designated m2-phage (amino acid sequence: *C-DRRDLPDWAIRA-C*). Figure 2 depicts a semi-quantitative dot blot analysis where phages are titrated and normalized for equal concentrations. In this manner, comparison of binding activities between different phages and a common target gp120 can be made. Clearly, m2-phage is a marked improvement over m1-phage and an intermediate phage 2A6. This is seen for both general binding to gp120 CDC451 and the induction of CD4i epitopes as detected by CG10 binding. As illustrated, this improvement is the result of only three compositional modifications. It would appear that slight reduction of the positive charge enhances the affinity for gp120, as the loss of lysine at position 10 of the peptide is common for both improved versions.

**Table 1:** Sequences of affinity-selected phages from gp120 CDC451 screens

**standard**		**stringent**
*CD**R**R**DLP**Q**WA**IRE**C**	**C**A**R**S**DLP**L**WA**KRE**C**	**CD**R**R**DLP**D**WA**IRA**C**
**C**D**R**S**DLP**Q**WA**TSV**C**	**C**D**R**K**DLP**E**WA**KRE**C**	**C**D**R**R**DLP**E**WA**LRA**C**
**C**D**R**R**DLP**Q**WA**ETV**C**	**C**D**R**R**DLP**E**WA**KRE**C**	**C**A**R**S**DLP**E**WA**NRA**C**
**C**D**R**R**DLP**Q**WA**NRA**C**	**C**D**R**L**DLP**Q**WA**NRA**C**	**C**D**R**R**DLP**Q**WA**VSA**C**
**C**G**R**R**DLP**K**WA**MRE**C**	**C**D**R**S**DLP**Q**WA**ISA**C**	**C**D**R**R**DLP**Q**WA**KEV**C**
**C**D**R**N**DLP**Q**WA**KSA**C**	**C**D**R**R**DLP**Q**WA**ISA**C**	
**C**E**R**R**DLP**Q**WA**MSV**C**	**C**D**R**R**DLP**Q**WA**ISV**C**	
**C**E**R**S**DLP**Q**WA**ISV**C**	**C**D**R**R**DLP**Q**WA**LSA**C**	
**C**D**R**S**DLP**Q**WA**TRA**C**	**C**D**R**R**DLP**Q**WA**MSA**C**	

The biased random mutagenesis library was screened using standard or stringent conditions as indicated (see Methods). Hundreds of potential gp120 CDC451 binding clones were obtained and confirmed for gp120 binding, the sequences of a collection of which are given above. *The sequence of 2A6 peptide. **The sequence of m2-peptide (see Figure 2). For convenience, the constant first and last cysteine residues as well as residues of a common motif (xRxDLPxWAxxx) are shown in bold.

To further confirm that the sequence of m2-peptide is specific for its binding activity to gp120, this binding was compared to a scrambled version of the same composition yet different linear sequence (*C-DLWRIRADRAPD-C*, note the flanking cysteine residues were maintained to ensure a disulfide looped conformation, see Figure 2).

**Figure 3:**
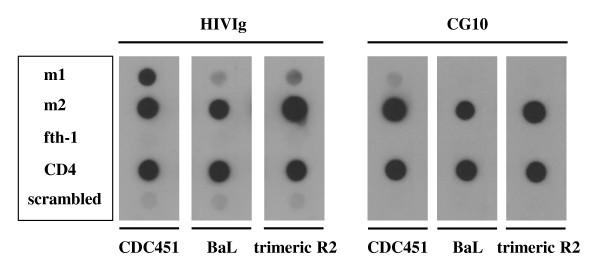
**The m2-phage binds monomeric and trimeric envelope proteins of different HIV-1 isolates.** Equal amounts of phages and CD4 were applied to nitrocellulose filters and incubated with gp120 CDC451, gp120 BaL, trimeric R2 gp140 or without any envelope as indicated (no signals developed in the absence of envelope, not shown). Captured envelope proteins were detected using HIVIg or the CD4i mAb CG10.

Next, we tested whether or not m2-peptide had acquired the ability to bind and induce conformational changes in envelopes which have been proposed as potential vaccine candidates, such as gp120 from the BaL isolate of HIV-1 [36,37,46] as well as trimeric gp140 [47,48]. As can be seen in Figure 3, m2-phage, compared to m1, indeed gained the ability to bind gp120 from the primary BaL isolate as well as trimeric R2 gp140, and was able to elicit CG10 binding in both.

**Figure 4:**
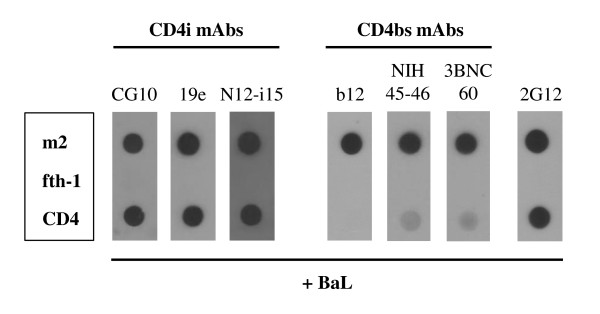
**The CD4 binding site is not compromised by m2-phage binding.** The m2 and fth-1 phages were applied to nitrocellulose filters along with CD4. The filters were incubated with gp120 BaL or without gp120 (no signals developed in the absence of gp120, not shown) and probed with three stringent CD4i mAbs and three CD4bs mAbs as indicated. Binding is also illustrated for mAb 2G12 which recognizes a mannose-rich epitope on gp120. Note that m2-phage induces binding for all three of the CD4i mAbs and does not interfere with the binding of the CD4bs mAbs. The binding of the CD4bs mAbs is sensitive to the capture of gp120 by CD4 as expected.

Figure 4 demonstrates the capacity of m2-phage to induce the binding of multiple stringent CD4i mAbs [14-17,25,38-40] as well as enable the binding of three CD4bs mAbs [41,49,50] which represent different aspects of the complex CD4bs [41,51,52]. This latter observation is consistent with the conclusion that m2 binds a distinct and different epitope on HIV envelope as compared to CD4 and CD4bs mAbs. This was further substantiated by three independent lines of investigation:

#### Simultaneous binding of CD4 or CD4 binding site mAbs with m2-phage

**Figure 5:**
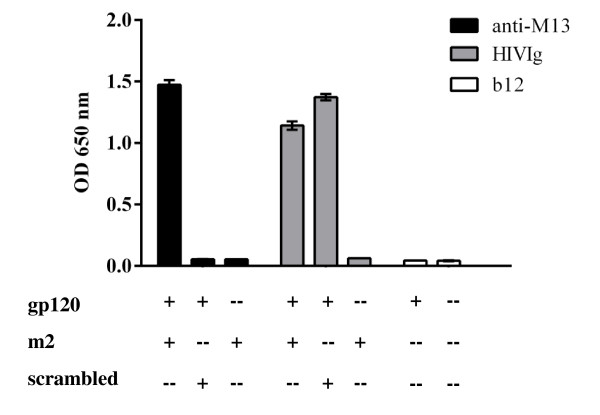
**The binding sites for CD4 and m2-phage are different.** CD4 was applied to ELISA wells and used to capture gp120 BaL. Subsequently m2-phage was added and detection of phage and gp120 was achieved with anti-M13 mAb, HIVIg or b12 mAb as indicated. Note that whereas CD4 capture inhibits b12 binding as expected, occupation of the CD4bs does not interfere with m2 binding. The experiment was carried out in duplicate.

**Figure 6:**
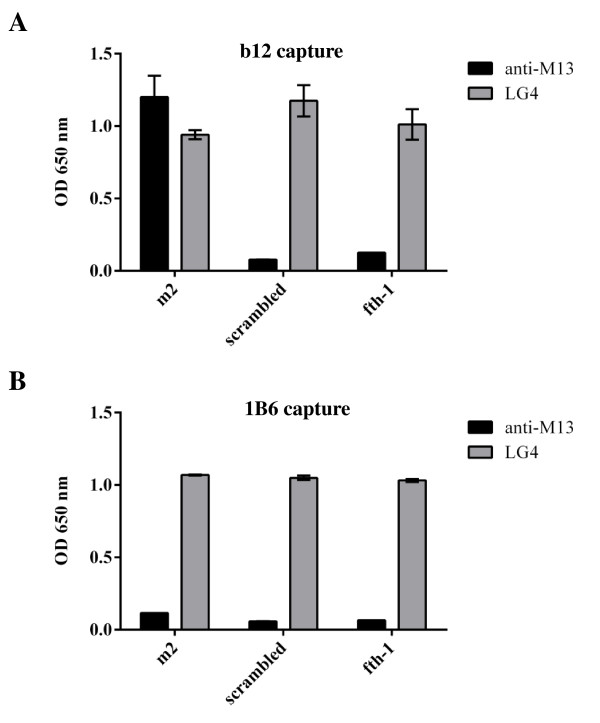
**Comparative capture of gp120 with two defining mAbs.** gp120 BaL was captured on ELISA wells using immobilized b12 mAb **(A)** compared with immobilized 1B6 mAb **(B)** and incubated with different phages as indicated. Bound phages were detected with rabbit anti-M13 polyclonal sera while detection of gp120 was accomplished using the LG4 mAb. Note that both b12 and 1B6 are efficient in capturing gp120 BaL yet distinct regarding overlap with the m2 binding epitope. The b12 epitope overlaps the CD4bs (see for example Figure 5) and does not interfere with m2 binding. The 1B6 epitope is different and distinct, competes for m2 binding and does not interfere with binding of either CD4 or b12 (see Figure 7). The experiment was carried out in duplicate.

In Figure 5, CD4 was plated onto ELISA wells and used to capture gp120 BaL. It is clearly shown that although the CD4bs is occupied, m2-phage continues to bind to the captured gp120. The fact that CD4 binding is the sole mechanism for gp120 capture is illustrated by the lack of mAb b12 binding in the same experiment. Likewise, this can be further supported when mAb b12 is used to capture the viral envelope protein (Figure 6A). Once again it is clearly shown that m2-phage is able to bind mAb b12-captured gp120 BaL. In fact, mAb b12 binding may be considered an even more demanding criterion than other neutralizing mAbs that target the CD4bs, as it requires a complementary pocket to accommodate W100 of its CDR3 [51] .

#### Binding of the 1B6 mAb

**Figure 7:**
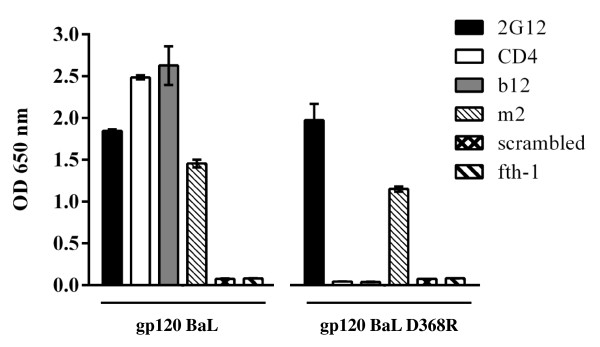
**Mutagenesis of the CD4 binding site does not alter the m2 binding site.** Wild type gp120 BaL or the gp120 BaL D368R mutant were captured onto ELISA wells using mAb 1B6 or HIVIg as indicated. The captured gp120 was then detected with various anti-gp120 mAbs (as indicated). CD4 binding to captured gp120 was detected with biotinylated anti-CD4 mAb CG9. Bound phages were detected with an anti-M13 mAb. Note the 1B6 mAb which competes with m2 binding (Figure 6) does not interfere with anti-gp120 mAbs or CD4. The experiment was carried out in duplicate.

Another line of support is found in using a different mAb, mAb 1B6, specific for gp120, which does not interfere with mAb b12 or CD4 binding, yet selectively competes and inhibits m2-phage binding. Thus one concludes that the two epitopes, those of 1B6 and b12/CD4 are different and distinct (Figure 6B and Figure 7).

#### Point mutagenesis of the CD4 binding site

A third line of proof for allosteric binding of m2-phage compared to CD4 is given in Figure 7. Here we compared the binding of m2, CD4 and mAb b12 to gp120 BaL and D368R mutated gp120 BaL. As expected, the D368R mutant had no affinity for either CD4 or b12 [53-58] yet continued to bind m2-phage. Hence, the m2:gp120 complex differs structurally from that of CD4 complexed gp120, in that in the latter the CD4bs is occluded thus preventing the binding by b12 and other CD4bs mAbs. These CD4bs defining mAbs bind m2:gp120 complex as is demonstrated in Figures 4, 6 and 7.

Other truncations and modifications of gp120 which were recently implicated as important for the regulation of the CD4-bound state [59] (deletion of the V1, V2 and V3 loops) do not affect CD4 binding or m2 binding as well (not shown), indicating that the m2 binding site, like that of CD4, does not require either of these variable loops.

### Attempts to further optimize the m2-peptide

**Figure 8:**
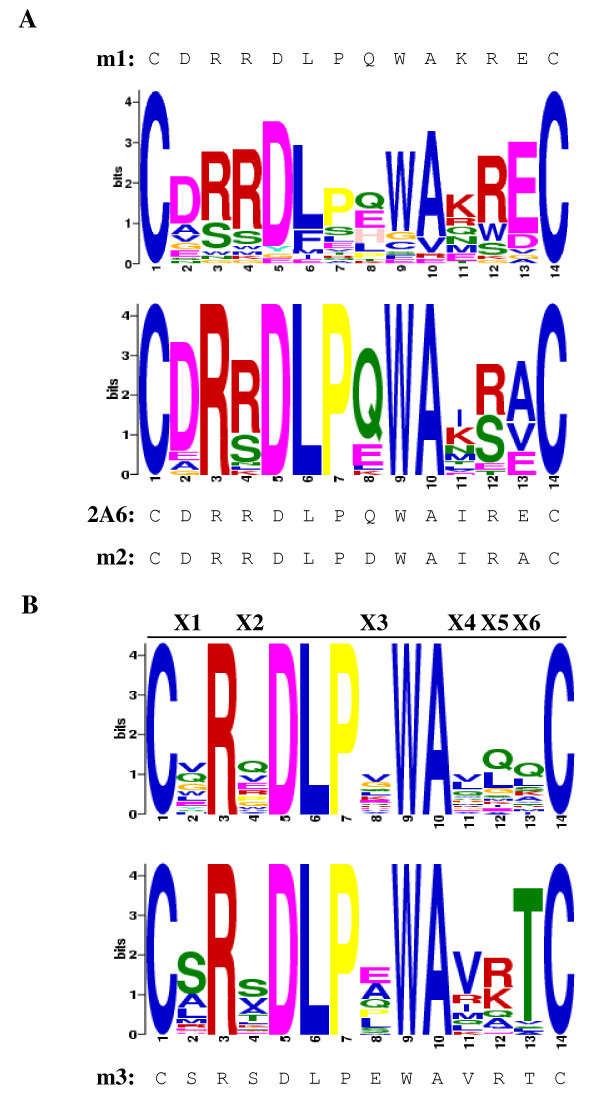
**Analysis of gp120 CDC451-binding peptides affinity selected from the biased random mutagenesis library and analysis of the “X6 NNK” m2-based phage display library. A.*Analysis of the biased random mutagenesis library.*** 24 randomly selected phages from the biased random mutagenesis m1-based library and 23 affinity selected phages obtained after screening of the biased random mutagenesis m1-based library against gp120 CDC451 were sequenced. The sequences were used as input for the MEME Suite motif analysis software [60,61] and two logos were produced (random peptides top and affinity-selected peptides bottom). The sequences of the m1, 2A6 (standard screening) and m2 (stringent biopaning) peptides are provided for comparison. **B.*Analysis of the “X6 NNK” m2-based library.*** Logos were prepared from the sequences of 20 randomly sampled phages of the “X6 NNK” m2-based library (upper logo) and those of 41 affinity selected phages obtained by screening the library with envelope as described in the text (lower logo). The sequence of m3-peptide is provided for comparison.

The transition from m1-peptide (*C-DRRDLPQWAKRE-C*) to an improved version, m2-peptide (*C-DRRDLPDWAIRA-C*), was accomplished by construction and stringent screening of a randomly mutated peptide library based on the m1-peptide (Figure 8A, upper logo). Examination of the sequences obtained from the screens revealed a core motif of six residues common to all of the peptides that continued to demonstrate induction of the CD4i conformation (Figure 8A, lower logo). The fact that these residues were specifically selected over a number of amplification rounds, and in different screens suggested that this core motif was functionally important. The other six residues, however, appeared to be less conserved, although some strong tendencies were found (for example a preference for Q < E < L < K is identified at position 8). It was assumed that the six core residues are essential for specific gp120 binding, while the other six variable positions allowed some room for further “adjustment” of the binding interaction in terms of strength and conformation modulation. The existence of a definite consensus motif and variable positions which might afford improvement of binding justified the production of a third library for further optimization. Hence we constructed the “X6 NNK” m2-based library in which the six consensus residues were left unchanged, while the other six positions were allowed to assume all possible amino acid residues (see Figure 8B, upper logo).

The “X6 NNK” library (complexity = 10^8^ variants) was screened against both monomeric gp120 BaL and trimeric R2 gp140 under various conditions designed to select for improved binders (see Methods). A total of over 3500 clones were selected for BaL/trimeric R2 binding or directly for CG10 induction, of which over 70 clones were further characterized and sequenced (see Table 2). The structural tendencies derived from this analysis are given in the motif in Figure 8B, lower logo.

**Table 2:** Sequences of peptides isolated by screening the “X6 NNK” m2-based library against gp120 BaL and trimeric R2 gp140

**sequence**	**monomer**	**trimer**
**C**S**R**S**DLP**E**WA**VRT**C**	15	13
**C**A**R**V**DLP**L**WA**VKT**C**	1	2
**C**E**R**S**DLP**A**WA**IKT**C**	3	0
**C**S**R**A**DLP**A**WA**VKT**C**	1	1
**C**S**R**K**DLP**S**WA**VKT**C**	2	0

The “X6 NNK” library was screened using standard conditions, stringent conditions or the complexed bait method, see Methods. Thousands of potential gp120 BaL binding clones and trimeric R2 gp140-binding clones were obtained of which more than a hundred were confirmed as envelope binders. A total of 75 clones were sequenced and the sequences of peptides which were isolated against both envelopes or isolated more than once are shown (the number of times each sequence was isolated for each envelope is given in the table). For convenience, the constant first and last cysteine residues, as well as residues of the X6 common motif, (xRxDLPxWAxxx) are shown in bold. Moreover, note the common Threonine at position 13.

Clearly, irrespective of whether the screening was performed using monomeric or trimeric envelope, and stringent or relaxed conditions one unique peptide was isolated at a preferred frequency as compared to the other peptides selected. This peptide, *C-SRSDLPEWAVRT-C* (designated m3), was tested for binding and compared to m1 and m2. Semi-quantitative dot blot analyses (see Methods) indicate preferred binding of gp120 by m3 *vs.* m2 and higher ability to induce CG10 binding (not shown).

**Figure 9:**
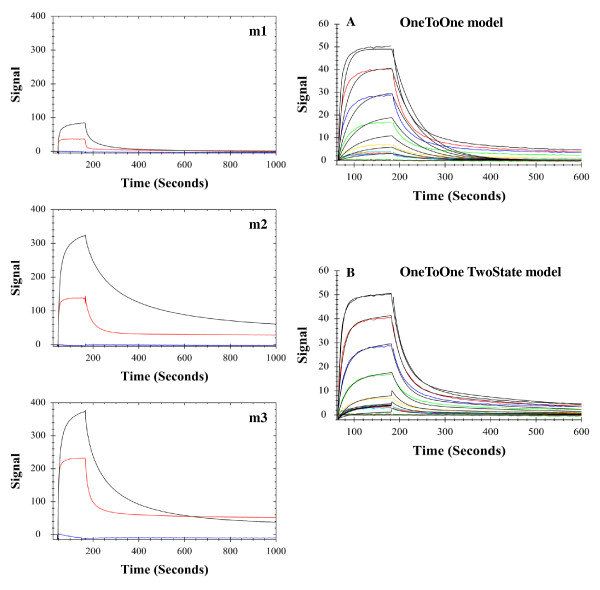
**SPR analyses of gp120 CDC451 binding to peptides.** Biotinylated phages displaying peptides m1, m2 and m3 were immobilized on CM5 sensor chips coated with streptavidin and reacted with either gp120 (red), mAb CG10 (blue) or with the mixture gp120 + CG10 (black). Y-axes (Signal) were adjusted according to baseline. As can be seen, the binding to m2-phage is markedly improved as compared to m1. The m3 peptide binds marginally better than m2. A scrambled peptide which was also tested in this setting did not show any binding to gp120 or CG10 (not shown). The two right-hand panels depict binding kinetics of two fold serial dilutions of gp120 (3.9-250nM) to the immobilized m2 displaying phage. The experimental data (colored curves) were fitted (black curves) using TraceDrawer 1.5 software (Ridgeview Instruments AB Uppsala, Sweden). As can be seen in A the OneToOne model does not fit the data very well (Chi^2^ 4.25). Using the OneToOne TwoState model the fit is markedly improved (Chi^2^ 0.32) supporting the conclusion that binding of m2-phage to gp120 is associated with conformational rearrangements.

In order to further quantify and compare binding activities and inductive potentials for m1, m2 and m3 peptides, we conducted biophysical analyses using surface plasmon resonance (SPR) as is shown in Figure 9.

Binding assays for m1, m2 and m3 (Figure 9) demonstrate that all three phage displayed peptides have no affinity for CG10, that they bind gp120 CDC451 and that this binding induces CG10 binding as well. Comparing the sensorgrams clearly illustrates m3 < m2 < m1 binding of the three peptides tested. We then attempted to conduct kinetic measurements of the binding for the three peptides. Generally, the classical OneToOne model fit poorly with the data in these experiments (see for example Figure 9A). Therefore, we tested an alternative model of binding, the OneToOne TwoState model, used in cases where conformational change might be involved [62,63], and found that this model fits the data very well (Figure 9B). Thus, for example, the Chi^2^ values for m1 were 2.91 and 0.94 for the OneToOne compared to the OneToOne TwoState models respectively. This same better fit was measured for m2-phage as well (Chi^2^ 4.25 compared to 0.32, compare Figure 9A with Figure 9B). From these data, affinity constants for m1 and m2 of 199.0 nM and 109.0 nM respectively can be calculated. The fit for m3, however, although improved (Chi^2^ 158 compared to 10.08) using the OneToOne TwoState model, was not as good. Therefore, the calculated affinity (8.75 nM) must be regarded with caution.

Thus, we have been able to sequentially screen and optimize the lead m1-peptide to generate m2 and m3 through the production of targeted phage display libraries.

Comparison of the m1, m2 and m3 peptides for their binding to gp120 and CD4i induction illustrates that the major improvement is in the transition from m1 to m2, while moving on to m3-peptide had a less dramatic effect. Thus, the m2 and m3 peptides seem to closely approach the ultimate level of optimization obtainable using functional screening of mutant libraries.

## Discussion

The use of random or customized peptide libraries for the isolation of gp120 binders has been reported in a number of previous studies, all of which with the purpose to isolate viral entry antagonists for therapeutic applications [64-66].

The goal in this study, however, is completely different. The objective was not to identify novel peptides that broadly cross react with a diversity of HIV isolates and inhibit their entry. Quite the contrary, our intent was to discover peptide modulators that better expose critical epitopes of HIV. In fact, our first lead peptide, m1-peptide, had very restricted binding and, for the most part, was specific for the HIV strain used as bait.

The criterion for selection of m1-peptide was the ability of gp120 to acquire recognition by stringent CD4i antibodies (i.e., CG10) following peptide binding. In accordance, upon binding to gp120, the m1-phage induced the epitopes recognized by the mAb CG10 in the absence of CD4 and was further pursued because of this intriguing characteristic. Whereas initially it might have been expected that the m1-phage functioned as a CD4 mimetic, we discovered that m1-phage is able to elicit the CG10 epitopes allosterically by binding to a distinct, and as yet unknown, surface on gp120. In doing so, m1 and its optimized derivatives cause changes in the orientation of the core backbone so as to stabilize the CD4i epitopes associated with the bridging sheet and V2 loop, as indicated by the acquired binding of three stringent CD4i mAbs CG10, 19e and N12-i15 respectively, as well as by the binding of relaxed CD4i mAbs 17b [9,11] and 48d [8] (not shown).

Optimization and production of next generation peptides were conducted so to improve the m1-peptide, viz. to bind additional HIV envelopes at higher affinity and ability to induce CG10 binding. For this, two custom tailored libraries were constructed and screened using various panning conditions leading to the isolation of peptides m2 and m3. Whereas m2 is dramatically more efficient than was m1-phage, m3-phage seems to be only marginally improved, indicating that we reached a limit in our ability to improve this peptide using the methods described.

These peptides were intended to alter the conformation of a given envelope with the strategic goal of developing an immunogen. Hence, in the transition from m1-peptide to its derivatives, we aimed to optimize peptide binding to a recognized vaccine candidate, in this case gp120 BaL (which has been used in a number of immunization studies [36,37,46]) as well as the trimeric gp140 from the R2 isolate proposed for vaccine development by Zhang et al. [48]. Other immunogens are being considered such as JR-FL [67,68], an HIV-1 global consensus envelope CON-S [69,70] and gp140 from clade C [71]. Preliminary binding studies found that m2 and m3 (but not m1-peptide) bind these alternative vaccine candidates to various degrees (data not shown).

Therefore, peptides m2 and m3 represent an additional reagent in the tool box of ligands designed so as to stabilize gp120 into a preferred CD4i conformation.

Generally, a number of approaches have been taken in the past so to stabilize gp120 in a CD4i conformation, the first based on production of stable complexes of gp120 and CD4 [35-37,46,72-75]. Fouts et al. reported that chemically cross-linked gp120:CD4 complexes raised antibodies which could neutralize primary viruses regardless of co-receptor usage and subtype in primates, while anti-gp120 sera only neutralized T-cell lab adapted strains [36]. The implementation of the gp120:CD4 complex culminated in the development of the FLSC complex comprised of gp120 BaL and CD4 (D1D2) linked *via* a flexible linker [46]. Following SHIV-challenge, FLSC-vaccinated monkeys showed a stronger anti-CD4i response that correlated with an enhanced decline and clearance of plasma viremia and absence of tissue viremia compared to unvaccinated controls, thus establishing a connection between CD4i antibodies and protection from disease [37]. The second approach towards stabilization of CD4i epitopes is exemplified by studies in which the CDR2-like domain of CD4 was reproduced on a stable and permissive scaffold in the form of scyllatoxin thus yielding a CD4-mimetic [10,76-78]*.* Several generations of such CD4-mimetics were evaluated to define the optimal molecule for use in complex with the HIV-1envelope as a vaccine, the latest “miniCD4” (M64U1-SH) being incorporated in a stable cross-linked complex with oligomeric gp140 [79]. Immunization of rabbits with this complex resulted in antibodies that neutralized heterologous Tier 1 viruses and HIV-2 (in the presence of sub-inhibitory concentrations of CD4) as compared to antibodies from gp140-vaccinated rabbits, indicating the selective elicitation of CD4i antibodies, whereas rabbits immunized with gp140 developed mostly anti-V1V2/V3 and anti-CD4bs antibodies.

Alternatively, a partial CD4-bound state of gp120 might be stabilized by limiting the conformational flexibility of gp120 through strategically placed cavity-filling mutations and addition of inter-domain disulfide bonds [41]. For example, Xiang et al. introduced the S375W mutation to the gp120 YU2 core so as to fill-in the “Phe43” cavity of gp120 and showed that this single mutation partially stabilized a CD4-bound conformation of gp120 [12]. Since this mutant was impaired in b12 recognition, a second mutation, T257S, was required in order to restore b12 binding [80]. Rabbits immunized with the double mutant completed with a number of stabilizing inter-domain disulfides developed strong anti-CD4i responses [81].

Exposure of the CD4i epitopes, using the actual CD4 molecule or a CD4-mimetic, targets the vicinity of the CD4bs *per se* and so the resulting antigens are devoid of a functional CD4bs. Though stabilization of a partial CD4-bound conformation through alteration of the amino acid composition of the gp120 does not physically obscure the CD4bs, demonstrating it to be functional to some degree, these stabilized cores elicit anti-CD4bs antibodies less efficiently than unmodified cores [81]. Obviously, the CD4bs is of great immunological importance as demonstrated by mAb b12 [41,49] and the recently isolated anti-CD4bs BCN antibodies such as VRC01 [82,83], VRC-PG04 [84], NIH45-46 [50].

The peptides reported here are distinct in the sense that whereas they induce the exposure of the CD4i epitopes, they do so allosterically, thus creating an unmodified gp120 (devoid of mutations) stabilized in a CD4i conformation, as indicated by its ability to bind the most stringent CD4i mAbs (CG10, 19e and N12-i15) as well as a more relaxed panel of CD4i antibodies (such as mAbs 17b and 48d).

Evidence that the m2 sequence *per se* binds gp120 (independently of residues that might be contributed by the phage major-coat Protein 8 scaffold) was provided by fusing m2 to the N-terminus of the minor phage Protein 3. Here m2-Protein 3 bound to gp120, and induced CG10 recognition well. In light of these data, one might argue that production of soluble synthetic versions of m2 would be useful. Indeed, production of such synthetic peptides was attempted for both m1 and m2 peptides, yet with little success. The main problem encountered was that these synthetic peptides were rather insoluble, and only low affinity binding could be measured after solubilization in organic solvents. Nonetheless, the m2 synthetic peptide could be shown to compete for m2-phage, albeit only at micro molar concentrations (data not shown).

Although the binding site for the m2-peptide remains a mystery, it does not seem to overlap with critical neutralizing surfaces of gp120. Efforts to map the m2 binding site or that of mAb 1B6 are ongoing. However, what is clear is that the induced conformational rearrangements typical of the CD4-bound state can be achieved while retaining a fully exposed and accessible CD4bs. This has been demonstrated not only by CD4 binding to m2-complexed envelope but also by binding of a panel of defining neutralizing CD4bs mAbs including 3BNC60, NIH45-46 and b12 [41,49,50]. The capacity to bind the mAb b12 simultaneously with m2 would indicate that in addition to exclusion of the CD4bs from the m2 epitope, one can argue that the proximal aspect of the hairpin β20β21 of the bridging sheet is also not necessary for m2 recognition. The unique requirements for mAb b12 binding, as compared to other CD4bs mAbs, have been clearly illustrated by Duenas-Decamp et al. [51]. The binding of mAb b12 demands access to the pocket that complements the W100 residue of its extended CDR3 loop, a pocket of HIV-1gp120 bordered by residues 416–423 just preceding β20.

Ultimately, one would like to produce a single chain peptide:gp120 complex to be used as a vaccine as was developed for gp120:CD4 complex by Fouts et al. [46]. For this, it would be advantageous to rationally design a functional linker that would effectively orient the fused peptide such that it can bind its epitope on the gp120 surface. In the absence of a definition of the m2 binding site and an atomic structure for full length unliganded gp120, such linkers can only be derived empirically. Once this is achieved, one can propose that gp120 complexed with m2 or m3-peptide may thus provide a new vaccine modality which benefits from both worlds: the ability to display the desired highly conserved CD4i epitopes without having to forfeit the CD4bs. Moreover, peptide-bound gp120 avoids complications of potential CD4 autoimmunity.

## Conclusions

Two types of vaccine relevant surfaces of HIV-1 gp120 have been recognized, the CD4bs and the neo-epitopes generated or revealed when gp120 associates with its primary receptor CD4 (i.e., CD4i epitopes). Here we describe phage-displayed peptides m1, m2 and m3 that induce the CD4i epitopes albeit without occluding the CD4bs. Three lines of evidence have been provided to prove the allosteric nature of this effect. Hence, binding of m2-peptide to HIV-1 envelope is accomplished independent of the CD4bs. This provides a new modality for vaccine development, viz. a mechanism to lock the envelope into a CD4 bound state yet enable a vacant CD4bs for immunological interrogation.

## Methods

### Antibodies, CD4 and envelope proteins

The murine anti-gp120 mAb CG10, which stringently recognizes the CD4i conformation of gp120 [14,17,38,39], the murine anti-gp120 mAbs LG4 (targets a conserved epitope at the carboxy-terminus of gp120), 9G3 and 1B6 [85], the murine anti-CD4 mAb CG9 [14], the murine anti-M13 Y2D mAb [86] and the rabbit polyclonal anti-M13 serum were produced at Tel Aviv University. The human anti-gp120 mAb N12-i15, which stringently recognizes the CD4i conformation of gp120, was produced at the Institute of Human Virology, University of Maryland School of Medicine, Baltimore, MA, USA [15,25,40]. The b12 mAb is a human anti-gp120 IgG kindly provided by D.R. Burton of Scripps Institute, La Jolla, CA, USA [49,55,87,88]. The human anti-gp120 mAb 2G12 was kindly provided by H. Katinger, Plant Biotechnology Unit, Department of Biotechnology, BOKU, Vienna, Austria [89,90]. The human anti-gp120 mAb 19e was a kind gift from J. Robinson, Tulane University Medical Center, New Orleans, LA, USA [8,9,11] . The human anti-gp120 PVL mAbs NIH45-46 and 3BNC60 were a kind gift from M.C. Nussenzweig, Laboratory of Molecular Immunology, The Rockefeller University, New York, NY, USA [50]. HIVIg, a pool of anti-HIV Igs from 30 HIV-1 infected individuals, was obtained from Nabi, Inc. Rockville, MD, USA. The murine anti-M13 mAb was purchased from GE Healthcare Bio-Sciences AB, Uppsala, Sweden. Recombinant gp120 BaL protein was produced in HEK 293 T cells and affinity-purified by as previously described [46] at the Institute of Human Virology, University of Maryland School of Medicine, Baltimore, MA, USA. The CD4bs-deficient D368R mutation in gp120 BaL [53-58] was prepared using overlap PCR as described in [91]. The oligonucleotides used in the overlap PCR were as follows: N-terminal-FOR 5′-cgccgccagcggtcgtcagaagcttatgcccatggggtctctg-3′; middle-REV 5′-gccgccgctgctgtgcttg-3′; middle-FOR 5′-caagcacagcagcggcggccgccccgagatcgtgacccac-3′; C-terminal-REV 5′-ttataatatctagattatcttttttctctttgcaccac-3′ (Hy Laboratories Ltd., Israel). The resulting mutated PCR product was cloned into the pCDNA3 expression vector (Invitrogen, CA, USA) and produced in HEK 293 T cells as previously described [46]. Recombinant trimeric R2 gp140 was kindly provided by G.V. Quinnan and C.C. Broder, Uniformed Services University of the Health Sciences, Bethesda, MD, USA [47]. Recombinant gp120 CDC451 protein was purchased from Advanced BioScience Labs, MA, USA. Soluble recombinant CD4 protein (D1-D4 domains) was a gift from GlaxoSmithKline, King of Prussia, PA.

### Design and construction of phage display libraries

The phage display peptide libraries used in this study were constructed at Tel Aviv University as previously described based on the fth-1 vector [92,93] and consisted of 12-mer, cysteine-looped phage displayed random peptides. The biased random mutagenesis library was constructed implementing “biased random mutagenesis” [45] where every position in the original DNA sequence of m1-peptide was laced with a 10% mixture of the other three phosphoramidites (3.33% of each). The “X6 NNK” m2-based library was designed so as to fix six core residues while allowing all possible amino acids to be incorporated at the remaining six positions, thus yielding a library with the pattern C-X_1_RX_2_DLPX_3_WAX_4_X_5_X_6_-C. Briefly, for the construction of the libraries, two 5′ biotinylated oligonucleotides were used. The first contained the “library” sequence flanked by *BglI* sites compatible with the two *SfiI* cloning sites of the vector. The second oligonucleotide complemented the 3′ end of the first and was extended to “fill-in” the complementary strand using Klenow polymerase. The product was digested with *BglI*, the short biotinylated segments were removed with streptavidin-conjugated beads, and the insert in the flow through was collected and cloned into *SfiI* digested fth-1 vector. This ligation mix was used to electroporate MC1061 cells; for details see [92].

### Screening of phage-display peptide libraries - biopanning

The biopanning and amplification procedures were carried out as previously described [92]. For standard biopanning, 6-well or 96-well cell culture plates (Corning Inc. Life Sciences, Tewksbury, MA) were coated with 20 μg/ml of monomeric gp120 or trimeric R2 gp140 in Tris-buffered saline; 50 mM Tris–HCl pH 7.5, 150 mM NaCl (TBS). The wells were blocked with 0.25% gelatin in TBS (TBSG), washed briefly twice with TBS, then incubated overnight at 4°C with 10^11^ phages of the relevant phage display peptide library suspended in TBSG. Subsequently, the plate was washed and the bound phages were eluted with glycine-HCl pH 2.2 and neutralized with Tris–HCl pH 9.1. For high stringency screening, wells of a 96-well cell culture plate were coated overnight at 4°C with 5 μg/ml of monomeric gp120 BaL or 1 μg/ml of trimeric R2 gp140 suspended in TBS. All washes were carried out with TBS/0.05% Tween 20. For complexed-bait screening: 5 μg/ml of gp120 BaL pre-mixed with 2.5 μg/ml CD4 (ca. 1:1 molar ratio, 1 hr at room temperature) or 2.5 μg/ml of trimeric R2 gp140 pre-mixed with 1.25 μg/ml CD4 (ca. 1:1 molar ratio, 1 hr at room temperature) in TBS were used to coat the wells of a 96-well cell culture plate (Corning Inc. Life Sciences, Tewksbury, MA) overnight at 4°C. All washes were carried out with TBS/0.05% Tween 20. Three additional rounds of amplification and biopanning were carried out for each screen. In order to confirm envelope binding to affinity-selected phages, *E. coli* DH5αF’ were infected with the eluted affinity-selected phages and the bacteria were plated on LB with 20 μg/ml of tetracycline. Single colonies were picked and grown as mini-cultures in U-bottom 96-well cell culture plates (Corning Inc. Life Sciences, Tewksbury, MA). The plates were centrifuged to pellet the bacteria and supernatants were transferred to 96-well flat bottom plates (Greiner Bio-One GmbH, Germany) containing polyethylene glycol/NaCl solution; 33% PEG, 3.3 M NaCl (PEG/NaCl) to precipitate phages followed by another centrifugation step to pellet the phages. Phages were re-suspended in TBS, quantified and used in confirmatory dot blot analyses.

### Dot blot analyses

Dot blots were used in this study for qualitative and semi-quantitative solid phase immunoassays [94,95].

#### Qualitative dot blots

Phages or proteins as specified were applied to nitrocellulose membrane filters using a vacuum manifold (2x10^10^ phages/dot or 1 μg/dot of protein). The filters were blocked using 5% skim milk in TBS for 1 hr at room temperature. Incubations with envelope proteins were performed overnight at 4°C at a concentration of 2.5-5 μg/ml in 5% skim milk/TBS. Incubations with antibodies were carried out for 90 min at room temperature with 1–5 μg/ml of the antibody of interest dissolved in 5% skim milk/TBS. Incubations with HRP-conjugated antibodies 1:5000 dilution (0.2 μg/ml) (Jackson, West Grove, PA) were carried out for 45 min at room temperature in 5% skim milk/TBS. Between incubations, the filters were washed 5 times with TBS/0.1% Tween 20. Signals were developed using the enhanced chemo-luminescence (ECL) reaction (Rhenium, Israel).

#### Semi-quantitative dot-blots

For quantification of the binding of envelope proteins and selected mAbs, it was first necessary to calibrate and normalize the application of equal amounts of phages used in the dot blot. The titer of various phages was determined using a plaque assay [92]. Then similar amounts of phages were two fold serially diluted in TBS and applied *via* a vacuum manifold onto nitrocellulose membrane filters. The dot blots were processed as described above using rabbit anti-M13 polyclonal antibody. Signals were generated using HRP-conjugates and ECL. For quantification, the filters were processed using the ImageQuant TL image analysis software (GE Healthcare Bio-Sciences AB, Uppsala, Sweden), and the concentrations of each phage type at each dilution were measured. Replica filters were used to react the calibrated phages with HIV envelope, followed by incubation with either HIVIg or CG10 mAb, as indicated. The signals were determined using the ImageQuant TL image analysis software after ECL.

All dot blot experiments were repeated at least 2–3 times. The qualitative analyses were typically performed using duplicate dots.

### Enzyme-Linked Immuno-Sorbent Assay (ELISA)

The wells of EIA/RIA 8-well strips or 96-well EIA/RIA plates (Corning Inc. Life Sciences, Tewksbury, MA) were typically coated overnight at 4°C with 10 μg/ml of mAb of interest in TBS. The wells were blocked for 1 hr at room temperature with 5% skim milk/TBS. Incubations with phages were carried out for 1-2 hrs at room temperature with 2 × 10^10^ phages/well. Incubations with gp120, CD4 or pre-formed gp120:CD4 complex (ca. 1:1 molar ratio, 1 hr incubation, room temperature) were performed for 1 hr at room temperature, at a concentration of 5–10 μg/ml in 5% skim milk/TBS. Incubations with various Abs were carried out in 5% skim milk/TBS at a concentration of 2.5 μg/ml for 1 hr at room temperature. Incubations with 1:2500–1:5000 (0.2-0.4 μg/ml) of secondary HRP-conjugated antibodies (Jackson, West Grove, PA) were carried out for 45 min at room temperature in 5% skim milk/TBS. Between incubations; wells were washed 3 times with TBS/0.05% Tween 20. Finally, the wells were reacted with TMB/E ELISA substrate (Merck Millipore, Billerica, MA). Absorbance was measured at 650 nm using a micro-plate reader (BioTek, Winooski, VT, USA). All ELISA experiments were repeated at least 2–4 times and typically performed in duplicates (unless specified otherwise).

### Surface Plasmon Resonance (SPR) analyses of phage-displayed peptides

The binding of phage-displayed peptides to gp120 CDC451 was compared using a BIACORE T-200 (GE Healthcare Bio-Sciences AB, Uppsala, Sweden). Series S sensor CM5 chips (GE Healthcare Bio-Sciences AB, Uppsala, Sweden) were used to immobilize 1300 response units of streptavidin (Ornat Biochemicals & Laboratory Equipment ltd., Israel) by standard amine coupling in 10 mM sodium acetate pH 5.0 and then loaded with 300 response units of p3-biotinylated phages displaying different peptides (production of p3-biotinylated phages is described in detail in [96]). Flow cells with immobilized streptavidin were used as a blank. Experiments were carried out at 25°C in 20 mM sodium phosphate pH 7.0 (PBS) and the sensor chips were regenerated using 10 mM glycine-HCl pH 2.5 after each injection cycle. Typically, 1 μM of each analyte (CG10, gp120 or a mixture of gp120 + CG10) was injected over the immobilized phages for 2 min at a flow rate of 50 μl/min followed by a 15 min dissociation period.

Kinetic analyses of the affinity of gp120 CDC451 to each of the phage-displayed peptides were performed at a concentrations ranging between 3.9-250 nM, including blank cycles of zero concentration samples and duplicate non-zero concentrations. gp120 CDC451 was injected over the immobilized phages for 2 min at a flow rate of 30 μl/min followed by a 15 min dissociation period. Regeneration using 10 mM glycine-HCl pH 2.5 was performed between the cycles. The experimental data were globally fitted to either the OneToOne model or the OneToOne TwoState model in TraceDrawer 1.5 software (Ridgeview Instruments AB Uppsala, Sweden) [62,63]. The latter model is described in principle by the following equation: A + B = AB = AB*. In this model, the analyte (A) binds to the ligand (B) to form an initial complex (AB) and then undergoes subsequent binding or conformational change to form a more stable complex (AB*). Interaction measurements with different lengths of association (injection times of 30, 180 and 600 sec) were used to validate the applicability of the OneToOne TwoState model (see [62,63]). The dissociation phase observed was clearly dependent on the injection time, where an increase in the contact time between gp120 CDC451 and the ligand led to a decrease in the dissociation rate as expected.

## Abbreviations

CD4i: CD4 induced; mAbs: monoclonal antibodies; CD4bs: CD4 binding site; BCN: Broadly cross neutralizing; FLSC: Full length single chain; SPR: Surface plasmon resonance; ECL: Enhanced chemo-luminescence.

## Competing interests

The authors declare that they have no competing interests.

## Authors’ contributions

JMG, ARB and GD conceived the idea and designed the experiments. ARB and GD performed the experiments. GK and GKL contributed to the design of experiments and consulted on the use of some of the antibodies. JMG, GKL and ARB analyzed the data and wrote the paper. All authors read and approved the final manuscript.
